# Cytomegalovirus Exposure in the Elderly Does Not Reduce CD8 T Cell Repertoire Diversity

**DOI:** 10.4049/jimmunol.1800217

**Published:** 2018-12-12

**Authors:** Paul Lindau, Rithun Mukherjee, Miriam V. Gutschow, Marissa Vignali, Edus H. Warren, Stanley R. Riddell, Karen W. Makar, Cameron J. Turtle, Harlan S. Robins

**Affiliations:** * Molecular and Cellular Biology Graduate Program, University of Washington School of Medicine, Seattle, WA 98195;; † Herbold Computational Biology Program, Fred Hutchinson Cancer Research Center, Seattle, WA 98109;; ‡ Ben Towne Center for Childhood Cancer Research, Seattle Children’s Research Institute, Seattle, WA 98101;; § Adaptive Biotechnologies, Seattle, WA 98102;; ¶ Program in Immunology, Fred Hutchinson Cancer Research Center, Seattle, WA 98109;; ‖ Department of Medicine, University of Washington, Seattle, WA 98195; and; # Bill and Melinda Gates Foundation, Seattle, WA 98109

## Abstract

With age, the immune system becomes less effective, causing increased susceptibility to infection. Chronic CMV infection further impairs immune function and is associated with increased mortality in the elderly. CMV exposure elicits massive CD8^+^ T cell clonal expansions and diminishes the cytotoxic T cell response to subsequent infections, leading to the hypothesis that to maintain homeostasis, T cell clones are expelled from the repertoire, reducing T cell repertoire diversity and diminishing the ability to combat new infections. However, in humans, the impact of CMV infection on the structure and diversity of the underlying T cell repertoire remains uncharacterized. Using TCR β-chain immunosequencing, we observed that the proportion of the peripheral blood T cell repertoire composed of the most numerous 0.1% of clones is larger in the CMV seropositive and gradually increases with age. We found that the T cell repertoire in the elderly grows to accommodate CMV-driven clonal expansions while preserving its underlying diversity and clonal structure. Our observations suggest that the maintenance of large CMV-reactive T cell clones throughout life does not compromise the underlying repertoire. Alternatively, we propose that the diminished immunity in elderly individuals with CMV is due to alterations in cellular function rather than a reduction in CD8^+^ T cell repertoire diversity.

## Introduction

As we age, immune function declines, a phenomenon known as immunosenescence. Large-scale changes in both the innate and adaptive immune system enhance susceptibility to infections and diminish responsiveness to vaccines, leading to increased morbidity and mortality (1–4). Many of these changes are exacerbated by pathogens that lead to chronic or persistent infections like CMV (4–7). CMV is a widely prevalent herpesvirus that establishes a lifelong latent infection; in the United States, the age-adjusted CMV seroprevalence is >50% in individuals between the ages of 6 and 49 y old (8, 9). In the elderly, high CMV Ab titers have been linked to increased mortality (10, 11), and CMV seropositivity has been shown to reduce survival in a cohort of Swedish octa- and nonagenarians (12). A study in a cohort of elderly individuals from the U.K. demonstrated that CMV seropositivity was associated with an increase in cardiovascular deaths, which decreased life expectancy in this group by nearly 4 y (13). In contrast, in exceptionally healthy older individuals in the United States, high CMV Ab titers were not indicators of physical or cognitive impairment (14). The relationship between CMV serostatus and mortality is thought to be the result of the large CMV-specific T cell response that develops postinfection and maintains the virus in a latent state.

Over time, massive CMV-driven CD8^+^ T cell clonal expansions are thought to compound a decline in immune function (15, 16). CMV-specific memory T cells differentiate into T effector memory cells expressing CD45RA (T_EMRA_), which have limited proliferative potential and resistance to apoptosis (5, 17). These cells possess a late-differentiated Ag-experienced phenotype that does not undergo replicative senescence due to repeated stimulation (5, 18). The accumulation of apoptosis-resistant T_EMRA_ clones in the CMV-seropositive elderly is believed to compromise T cell repertoire diversity (19–21).

T cell repertoire diversity is defined as the number, frequency, and distribution of clones within the T cell repertoire, and its reduction has been shown to decrease the breadth of the immune response against a wide spectrum of epitopes in mice (22, 23). In the elderly CMV seropositive, the persistence of T_EMRA_ clones is hypothesized to exacerbate competition between both the naive and memory CD8^+^ T cell repertoires for homeostatic survival signals, perpetuating a reduction in the diversity of each T cell subset (4, 21, 22, 24). This loss of T cell clones, combined with an age-related decline in naive T cell production and polyfunctional T cell responses against new Ags, suggests a mechanism for the increased mortality observed among the CMV-seropositive elderly (2, 21, 25–27). However, it is important to note that previous methods, including V–J tracking and spectratyping, lacked the sensitivity and specificity to interrogate the underlying naive and memory T cell repertoires in CMV (15, 22, 28–30). To gain insights into the nature of the entire CD8^+^ T cell repertoire in the natural setting of immune aging and chronic stimulation by CMV, we combine flow cytometry and immunosequencing of the TCR β-chain (TCRβ) as a measure of the diversity of the T cell repertoire.

To characterize the effects of aging and CMV on the T cell repertoire, we surveyed millions of T cell clones from the repertoires of 543 subjects across a wide range of ages and observed that a small set of clones dominate the repertoires of CMV-seropositive individuals. When we specifically examined the CD8^+^ T cell repertoires of CMV-seropositive elderly, we found that the most numerous 0.1% of peripheral blood clones comprised the majority of classical Ag-experienced CD45RO^+^ memory T cells and CD45RA-revertant T_EMRA_ compartments. We were able to examine in detail the impact of CMV on the structure of the underlying repertoire of these elderly individuals and failed to find evidence of compromised repertoire diversity in the presence of CMV-induced clonal expansions. Overall, our data suggests that the space occupied by CMV-specific clones grows considerably in the CMV-seropositive elderly without affecting the rest of the repertoire and that the repertoire broadens to accommodate these large clonal expansions.

## Materials and Methods

### Experimental cohort and study approval

For the large cohort of 543 donors, frozen PBMC samples were obtained from the Fred Hutchinson Cancer Research Center Research Cell Bank biorepository of healthy bone marrow donors. All donors used met the medical criteria set by the national marrow donor programs. These samples were previously tested for CMV serostatus (31). For the aged cohort of eight donors (≥70 y), fresh blood samples were obtained from the Fred Hutchinson Cancer Research Center Prevention Center. For both cohorts, donor protocols were approved and supervised by the Fred Hutchinson Cancer Research Center Institutional Review Board.

### CMV serology

For the large cohort of donors, CMV serostatus was extracted from historical clinical records. For the eight elderly donors, serum samples were tested for the presence of CMV IgG Abs using standard clinical laboratory assays at the University of Washington Medical Center.

### EBV serology

For the eight elderly donors, serum samples were tested for the presence of IgM, IgG, and nuclear Ab using standard clinical laboratory enzyme immunoassays at the University of Washington Medical Center.

### Complete blood counts

For the eight elderly donors, complete blood counts as well as CD4 and CD8 T cell counts were performed by the clinical laboratory at the University of Washington Medical Center using standardized assays developed by the Department of Laboratory Medicine.

### Cell sorting

For the eight elderly donors, PBMCs were isolated from fresh blood samples using density gradient centrifugation with Ficoll (GE Healthcare). CD3^+^ T cells were enriched from PBMCs by immunomagnetic selection using CD3 MicroBeads (Miltenyi Biotec, Auburn, CA). Cells were stained in the dark for 15 min with the following anti-human Abs: CD45RO PE-Cy7 (BD Biosciences, San Jose, CA), CD3 Alexa Fluor 700 (BD Biosciences), CD62L-PE (BD Biosciences), CD45RA-allophycocyanin (BD Biosciences), CD8-Pacific Blue (BD Biosciences), CD4 allophycocyanin-Cy7 (BD Biosciences), and LIVE/DEAD Aqua fluorescent reactive dye (Invitrogen, Grand Island, NY).

CD8^+^ T cell subsets were isolated using the BD FACSAria cell-sorting system (BD Biosciences), including CD8^+^CD45RA^−^CD45RO^+^ (for CD8^+^ memory), CD8^+^CD45RA^+^CD45RO^−^CD62L^hi^ (CD8^+^ naive), and CD8^+^CD45RA^+^CD62L^lo/−^ (CD8^+^ T_EMRA_). FlowJo (TreeStar, Ashland, OR) analysis was used to determine the proportions of the different subsets as a fraction of total CD8^+^ T cells.

### Immunosequencing

For the large cohort, genomic DNA was extracted from PBMC samples using the QIAGEN DNeasy Blood Extraction kit (QIAGEN). An average of 2.5 μg of input DNA was used for sequencing each sample. For the aged cohort, total genomic DNA was extracted from sorted T cells using the QIAamp DNA Blood Mini Kit (QIAGEN). DNA could not be isolated from the CD4^+^ T cell sample from study subject four. At least 3.2 μg of input DNA was used for sequencing each population. For all samples, the CDR3 region of the rearranged TCRβ locus was amplified, sequenced, and processed using the immunoSEQ Assay (Adaptive Biotechnologies, Seattle, WA) (32–37).

### CMV stimulation

RV798 CMV-infected fibroblasts (38) from subjects four and five were used to stimulate autologous sort-purified CD8^+^CD45RA^−^CD45RO^+^ memory and CD8^+^CD45RA^+^CD62L^lo/−^ T_EMRA_ cells for 24 h. CMV-reactive T cells from each stimulated CD8^+^ T cell subset were sorted as CD8^+^CD137^+^ events (39). Memory and T_EMRA_ CD8^+^ T cells stimulated with uninfected fibroblasts were used as a negative control for CD137 expression. After stimulation, genomic DNA was extracted from sorted CD137^+^ memory and T_EMRA_ samples using the QIAamp DNA Blood Mini Kit (QIAGEN) and sequenced using the immunoSEQ Assay (Adaptive Biotechnologies).

### Repertoire diversity and clonality metrics

The Shannon entropy or diversity (*H*) is an index that combines measurements of species richness and abundance. For a sample with richness *S* and clone-wise population fractions given by *x*_1_, *x*_2_, …, *x_S_*, the Shannon diversity is defined the entropy of the clone-wise abundance distribution. The Shannon entropy favors neither rare nor dominant clones disproportionately because each clone is weighted by its frequency in the sample.

Clonality describes the degree to which expanded clones dominate the repertoire. The Shannon equitability (*E_H_*) is defined as *E_H_* = *H*/*H*_o_, where *H*_o_ is the maximum entropy and *H*_o_ = log *N*. Clonality is defined as (1 – *E_H_*) with larger values indicating more oligoclonal repertoires.

### Statistics

Nonparametric statistical tests were used throughout. Statistical tests were performed in R (http://www.R-project.org/). For multiple comparisons, Dunn z test approximation to a rank-sum test was used with the Benjamini–Hochberg method to control for multiple hypotheses (dunn.test package in R). For pairwise comparisons between the CMV-seropositive and -seronegative study subjects, a two-tailed Wilcoxon–Mann–Whitney rank-sum test was used (wilcox.test function in R). Spearman correlation coefficient was used for all correlations (cor.test function in R).

## Results

### Large clones compose a significant portion of the CMV-seropositive T cell repertoire

**FIGURE 1.**
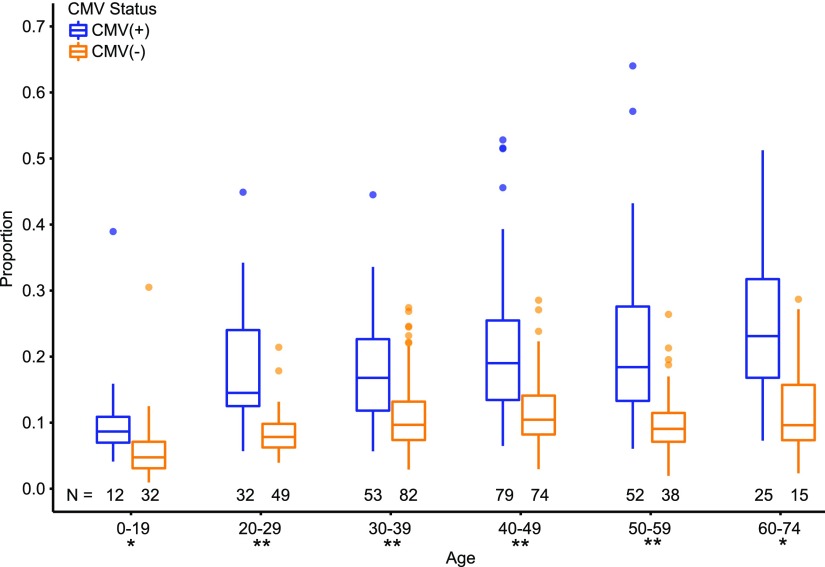
Impact of CMV on the proportion of large clones with age. Boxplot comparing the proportion of the most numerous 0.1% of clones in the peripheral blood T cell repertoire of CMV^+^ (blue) and CMV^−^ (orange) subjects. TCRβ sequencing was performed on the PBMCs of each subject. For each subject, the cumulative abundance of the most numerous 0.1% of clones was divided by the total sample abundance to yield a proportion. Nonproductive TCRβ rearrangements were excluded from this calculation. The band inside each box represents the median, and the whiskers extend to values that are within 1.5 times the interquartile range. Outliers are represented by dots. The N beneath each box represents the number of samples in each group. *p < 0.0005, **p < 0.0001.

To determine the impact of large T cell clones on the peripheral blood T cell repertoire, we examined the repertoires of 543 CMV-seropositive (CMV^+^) and CMV-seronegative (CMV^−^) subjects from a previous study (31) (see *Materials and Methods*). For each sample, large clones were defined as the most numerous 0.1% of clones (∼200 clones) in the peripheral blood T cell repertoire. Consistent with previous studies showing that CMV exposure induces massive T cell clonal expansions (40, 41), we found that the cumulative abundance of these large clones composed a significant proportion of the T cell repertoire in CMV^+^ compared with CMV^−^ individuals (*p* < 0.0005) in each age group (Fig. 1). Next, we investigated whether the cumulative abundance of the most numerous 0.1% of clones composed a greater proportion of the T cell repertoire with increasing age in either the CMV^+^ or CMV^−^. Except for comparisons to the youngest age group (significance threshold *p* < 0.02), we did not observe a significant age-related increase in the proportion of the peripheral blood T cell repertoire composed of these large clones. However, we did observe a weak but significant correlation in both the CMV^+^ (0.252, *p* = 4.2 × 10^−5^) and CMV^−^ (0.293, *p* = 2.8 × 10^−7^) between age and the proportion of the T cell repertoire composed of the most numerous 0.1% of clones. After early adulthood, the gradual age-related increase in the proportion of the repertoire composed of the largest clones in the CMV^+^ suggests that CMV-specific T cell clones stabilize with age.

### Large clones dominate the memory repertoires of the elderly

**FIGURE 2.**
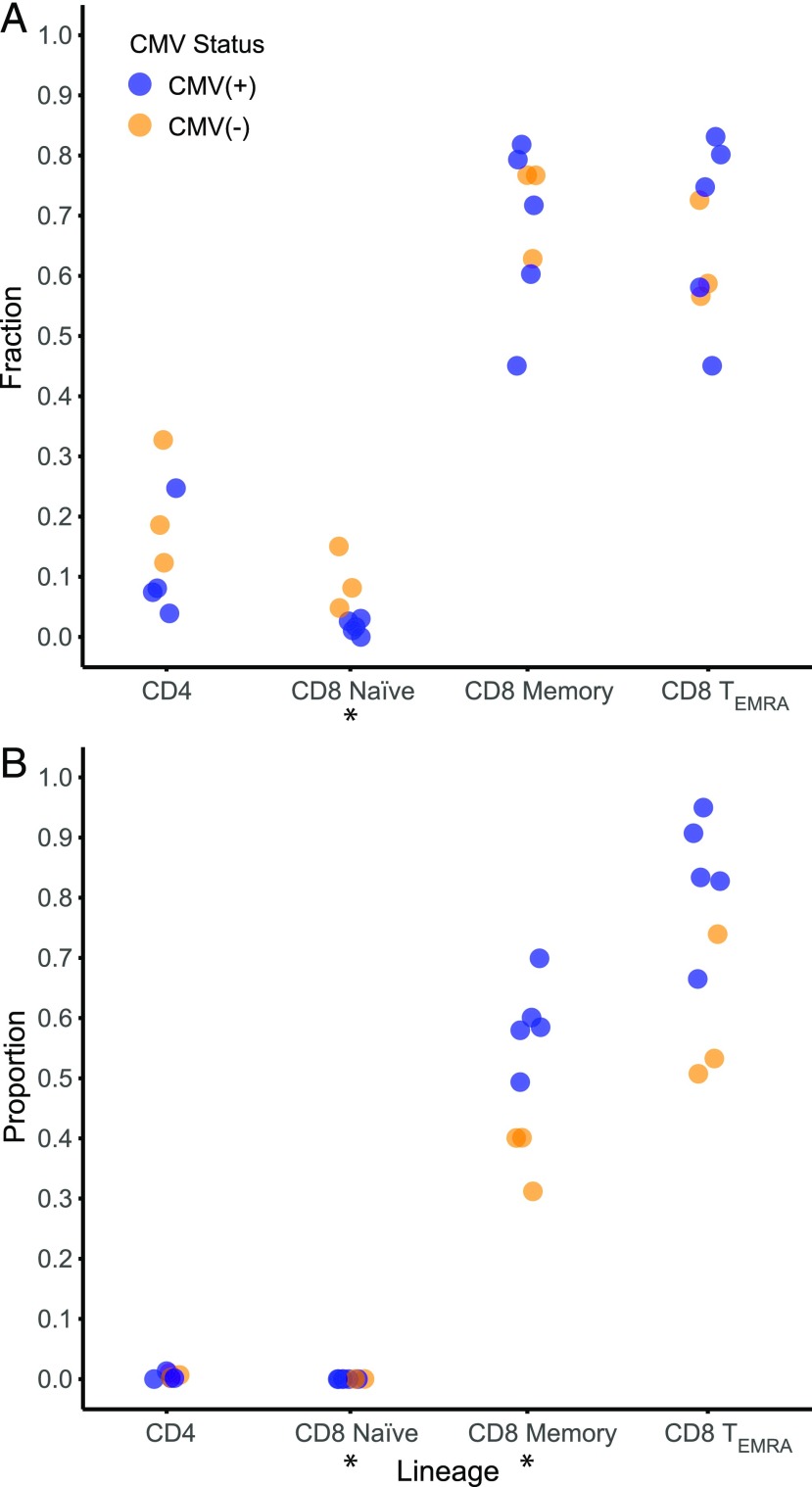
Distribution of large peripheral blood T cell clones in the elderly. (**A**) Scatterplot comparing the fraction of the most numerous 0.1% of peripheral blood T cell clones present in each sorted T cell subset in CMV^+^ (blue) and CMV^−^ (orange) subjects. TCRβ sequencing was performed on the PBMCs and specified T cell subsets of eight subjects >70 y old. Nonproductive TCRβ rearrangements were excluded from these analyses. The fraction of the most numerous 0.1% of peripheral blood T cell clones present in each T cell subset is depicted. (**B**) The proportion of each sorted T cell subset composed of the most numerous 0.1% of peripheral blood T cell clones in CMV^+^ and CMV^−^ subjects. The cumulative abundance of the most numerous 0.1% of peripheral blood T cell clones present in each T cell subset were divided by the total abundance of each subset to yield a proportion. The most numerous 0.1% of peripheral blood T cell clones found in both naive and memory or T_EMRA_ subsets were bioinformatically removed from the naive repertoire. *p < 0.05. CD4, Bulk CD4^+^ T cells; CD8 Memory, CD8^+^ central and effector memory T cells; CD8 Naive, CD8^+^ naive T cells.

To further investigate the effect of large T cell clones on the T cell repertoire of the elderly, we recruited five CMV^+^ and three CMV^−^ subjects between the ages of 70 and 74 (Supplemental Table I). We then isolated PBMCs as well CD4^+^, CD8^+^ naive, CD8^+^ memory, and CD8^+^ T_EMRA_ T cell subsets and examined the rearranged TCRβ DNA sequences in these samples (see *Materials and Methods*). As with the larger cohort, we observed that the cumulative abundance of the most numerous 0.1% of clones composed a larger proportion of the peripheral blood T cell repertoire in CMV^+^ subjects (*p* = 0.03) (Supplemental Fig. 1). We then analyzed the different T cell subsets to determine the lineage of the most numerous 0.1% of peripheral blood T cell clones. To minimize the effect of contamination during sorting, we bioinformatically removed from the naive repertoire the most numerous 0.1% of peripheral blood T cell clones that were also present in memory and T_EMRA_ samples from the same subject. Between 63 and 115 clones were removed from the naive repertoire of the different subjects. Unexpectedly, the fraction of the naive repertoire composed of these clones did not correlate with the purity of the sort (Supplemental Fig. 2). This observation could also be accounted for by our use of CD62L as opposed to CCR7 to divide the T cell subsets; however, CD62L was chosen because CMV-reactive clones are mostly CD62L^−^ (42), and CD62L and CCR7 are generally coexpressed (43). Therefore, it is likely that a combination of the sort criteria and the sort purity account for the presence of large peripheral blood T cell clones in naive T cell samples. Nevertheless, we found that the vast majority of the most numerous 0.1% of peripheral blood T cell clones resided in the CD8^+^ memory and CD8^+^ T_EMRA_ repertoires (significance threshold *p* < 0.01) (Fig. 2A). When we calculated the cumulative abundance of the most numerous 0.1% of peripheral blood T cell clones in each T cell subset, we observed that these large clones constituted a greater proportion of the memory repertoire in CMV^+^ compared with CMV^−^ individuals (*p* = 0.03) (Fig. 2B**)**. We also observed that a significant proportion of the naive T cell repertoire was composed of the most numerous 0.1% of peripheral blood T cell clones in the CMV^−^ compared with the CMV^+^ (*p* = 0.03), which is likely related to an increase in the homeostatic expansion of naive T cell clones in study subjects without CMV (44). The cumulative abundance of the most numerous 0.1% of peripheral blood T cell clones in the T_EMRA_ repertoires were not significantly different between the CMV^+^ compared with CMV^−^ despite the fact that in four of five CMV^+^ subjects these large clones composed >80% of the T_EMRA_ repertoire.

### Large clones are CMV reactive

**FIGURE 3.**
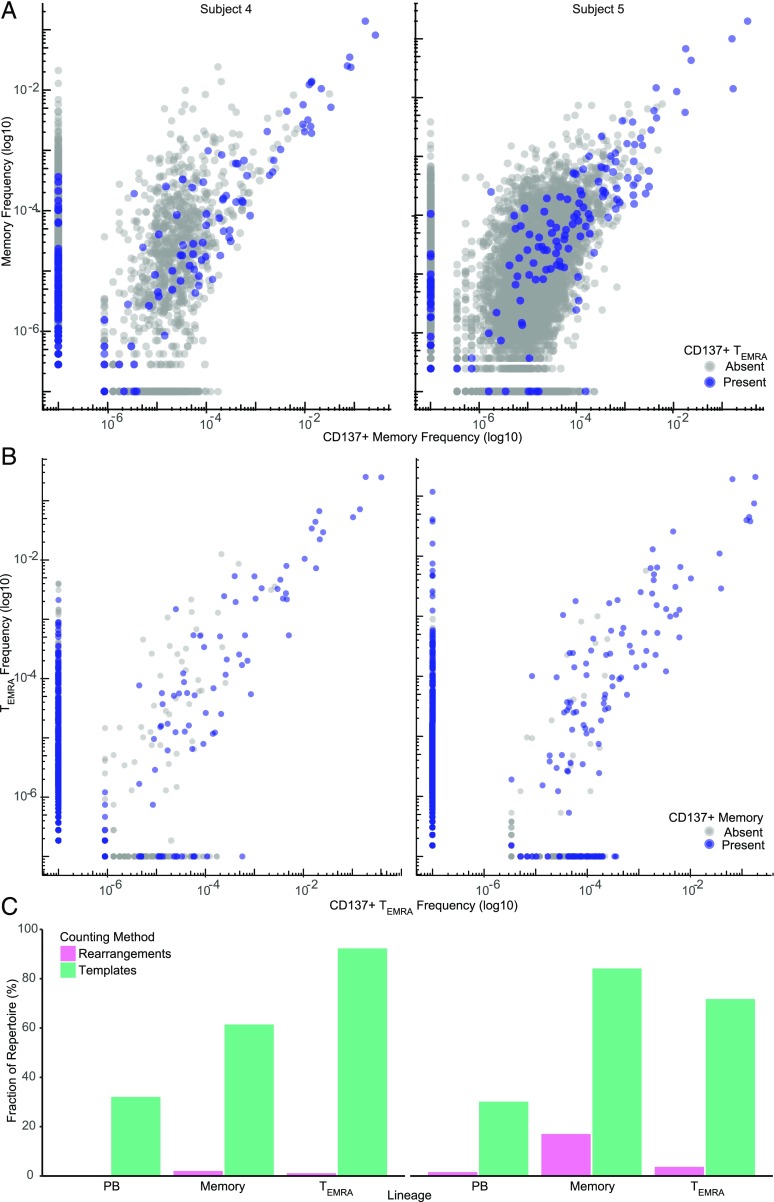
Identification of CMV-reactive T cell clones in the elderly. (**A** and **B**) Scatterplot comparing clone frequencies in CMV-stimulated CD137^+^ and resting memory (A) or T_EMRA_ (B) subsets from CMV^+^ subjects four and five. CD8^+^CD45RO^+^ memory and CD8^+^CD45RA^+^CD62L^lo/−^ T_EMRA_ cells were sorted and stimulated with autologous CMV-infected fibroblasts for 24 h. CD137^+^ T cells were then sorted and TCRβ sequencing was performed. The frequency of productive TCRβ sequences from unstimulated memory and T_EMRA_ samples are plotted against the frequency of productive TCRβ sequences from the corresponding CD137^+^ sample. Each point represents a unique clone. Points along the axis represent clones present in one sample. Points colored blue in (A) represent clones present in the CD137^+^ T_EMRA_ sample and in (B) represent clones also present in the CD137^+^ memory T cell sample. Logarithmic scale, base 10. (**C**) Comparison of the fraction of each T cell subset as unique rearrangements (pink) or reads (green) composed of CD137^+^ CMV-reactive clones. Memory and T_EMRA_ CD137^+^ TCRβ sequences were combined to capture all CMV-reactive clones present in each PBMC T cell repertoire. Pink represents the fraction of unique CMV-reactive clones found in each corresponding unstimulated sample. Green represents the cumulative abundance CMV-reactive clones found in each corresponding unstimulated sample divided by the total abundance of the sample. Memory, CD8^+^ central and effector memory; PB, peripheral blood.

To determine the proportion of the memory and T_EMRA_ repertoires dedicated to CMV, we isolated skin fibroblasts derived from CMV^+^ subjects four and five and infected them with CMV (see *Materials and Methods*). Memory and T_EMRA_ cells were then stimulated with these fibroblasts, and CD137^+^ activated T cells were sorted and sequenced. When we examined the relative frequency of CMV-reactive clones in each subject, we observed that, in both memory and T_EMRA_ subsets, the highest frequency clones were CMV reactive (Fig. 3A, 3B). Moreover, we found that the largest clones were present in both memory and T_EMRA_ subsets. Interestingly, in the T_EMRA_ population, we observed large clones in both subjects that did not express CD137 when stimulated with CMV (Fig. 3B). These large T_EMRA_ clones were also found in the CD137^+^ CMV-reactive memory subset, suggesting that phenotype contributes to CMV responsiveness. T_EMRA_ cells have previously been shown to contain CMV-reactive clones with limited proliferative potential and resistance to apoptosis (45). We then determined the cumulative abundance of CD137^+^ memory and CD137^+^ T_EMRA_ clones and calculated the proportion of PBMC, memory, and T_EMRA_ subsets allocated to CMV (Fig. 3C). We found that, in the two subjects studied in this manner, the 10 largest CMV-reactive memory clones composed 34.4 and 45.9% of the memory repertoire, whereas the 10 largest CMV-reactive T_EMRA_ clones made up 82.8 and 62.3% of the T_EMRA_ repertoire. We also observed that the proportion of the peripheral blood repertoire dedicated to CMV is very similar to the proportion of the repertoire composed of the most numerous 0.1% of T cell clones. Notably, 37.4 and 42.0% of the most numerous 0.1% of peripheral blood T cell clones were found to be CMV reactive in each of the subjects. Additionally, as a proportion, the cumulative abundance of CMV-reactive clones accounted for 75.4 and 78.0% of these large peripheral blood clones. Together, these results demonstrate that the largest clones in the peripheral blood T cell repertoire are CMV reactive and that they reside in the memory and T_EMRA_ subsets.

### Effect of CMV on underlying T cell repertoire diversity

**FIGURE 4.**
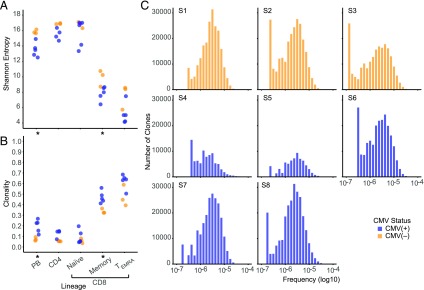
Effect of CMV on the underlying T cell repertoire. (**A** and **B**) Scatterplot comparing the Shannon entropy (A) or clonality (B) of each T cell subset in CMV^+^ (blue) and CMV^−^ (orange) subjects. The most numerous 0.1% of peripheral blood T cell clones found in both naive and memory or T_EMRA_ repertoires were bioinformatically removed from naive T cell entropy and clonality calculations. Nonproductive TCRβ rearrangements were excluded from these calculations. (**C**) Histogram comparing the frequency distribution of naive T cell clones in CMV^+^ and CMV^−^ subjects. Each bar represents the total number of unique clones present at a particular frequency. Naive T cell clones with frequencies greater than 10^−4^ are not displayed. Logarithmic scale, base 10. *p < 0.05. Memory, CD8^+^ central and effector memory; PB, peripheral blood. S, subject.

Considering that a significant proportion of the repertoire is dedicated to CMV, we next sought to determine the effect of large clones on the underlying T cell repertoire. We calculated the Shannon entropy and clonality metrics for each T cell subset under study (see *Materials and Methods*, Fig. 4A, 4B). To remove the possible confounding effect of the purity of the cell sort, we bioinformatically removed from the naive repertoires of each participant the most numerous 0.1% of peripheral blood T cell clones also found in the memory and T_EMRA_ subsets. As expected, we observed that the CD4^+^, CD8^+^ naive, and peripheral blood T cell repertoires of all subjects are significantly more diverse as compared with the memory and T_EMRA_ subsets from the same individuals (significance threshold *p* < 0.01). When we compared CMV^+^ and CMV^−^ individuals, we found that both the Shannon entropy and clonality of the CD4^+^, CD8^+^ naive, and CD8^+^ T_EMRA_ repertoires were not significantly different. However, the peripheral blood and memory repertoires of the CMV^+^ were less diverse compared with the CMV^−^ (*p* = 0.03). These results demonstrate that the decrease in overall T cell repertoire diversity in the CMV^+^ is due to a decrease in diversity of the memory T cell subset. To determine whether CMV altered the structural characteristics of the naive repertoire, we examined the distribution of low-frequency clones in the naive repertoires of each subject. We found nearly identical clone frequency distributions between CMV^+^ and CMV^−^ individuals (Fig. 4C). Although naive T cells make up a smaller fraction of the total CD8^+^ T cell population in CMV^+^ subjects (Supplemental Table I), the overall structure of the repertoire appears to remain unmodified.

### The CD8^+^ repertoire expands to accommodate large clones

**FIGURE 5.**
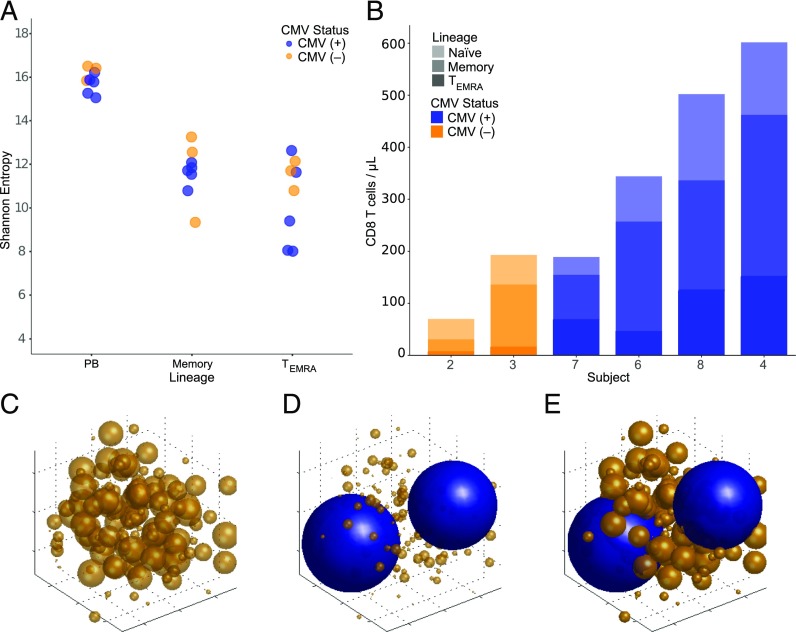
Accommodation of large clonotypes in the T cell repertoire. (**A**) Scatterplot comparing the Shannon entropy in CMV^+^ (blue) and CMV^−^ (orange) subjects after removal of the most numerous 0.1% of peripheral blood T cell clones from each T cell subset. The most numerous 0.1% of peripheral blood T cell clones were bioinformatically removed from each T cell subset and the Shannon entropy values were recalculated. Nonproductive TCRβ rearrangements were excluded from these calculations. (**B**) Stacked bar chart comparing the total number of T cells in each subset in four CMV^+^ and two CMV^−^ subjects. Clinical laboratory CD8^+^ T cell counts were performed on blood samples from six to eight study subjects. The relative frequency of each CD8^+^ T cell subset obtained by flow cytometry is used to identify the number of T cells in each subset. Light gray, Naive T cells; Medium gray, Memory T cells; and Dark gray, T_EMRA_ cells. (**C**–**E**) A model of the T cell repertoire with subordinate clones (orange) and CMV-reactive clones (blue) depicting the potential impact of CMV infection in (C) T cell repertoire at baseline prior to CMV exposure. All clones share similar frequency distributions. (D and E) T cell repertoire after CMV exposure with massive clonal expansions that supplant rare T cell clones from the repertoire (D) or that does not modify the underlying T cell repertoire (E). Sphere size corresponds to clone abundance. Memory, CD8^+^ central and effector memory; PB, peripheral blood.

To assess whether the differences in peripheral blood, memory, and T_EMRA_ repertoire diversities were the result of large CMV-driven clonal expansions, we removed the most numerous 0.1% of peripheral blood T cell clones from the peripheral blood, memory, and T_EMRA_ repertoires of each subject. When we recalculated the Shannon entropy, we found that the peripheral blood, memory, and T_EMRA_ repertoire diversities were indistinguishable based on CMV serostatus (Fig. 5A). Removing the most numerous 0.1% of peripheral blood T cell clones increased repertoire diversity in all subjects, suggesting that the presence of large clones does not dramatically affect the clonal composition of the underlying repertoire.

To reconcile the observation that very large CMV-reactive clones dominate the repertoire of elderly, CMV^+^ subjects without altering its underlying diversity and structure, we obtained diagnostic-quality T cell counts from six of the subjects in this study (see *Materials and Methods*). We then used relative frequencies of CD8^+^ T cell subsets obtained using flow cytometry to calculate the number of cells present in each subset (Fig. 5B). Interestingly, we observed that the CD8^+^ T cell repertoire broadens to accommodate large, CMV-fueled clonal expansions. This observation contrasts with conclusions from previous studies suggesting that large clonal expansions expel smaller clones from the repertoire to maintain homeostasis. Instead, our data suggests that the number of cells in the T cell repertoire increases over the lifespan of a subject to accommodate new Ag exposures as well as sustain a stable population of large clones that react to chronic viral infections.

## Discussion

In this study, we define the effect of CMV on the diversity and clonal structure of the aging immune system. Over time, chronic CMV infection sustains large CMV-specific clones in the circulating T cell repertoire (41, 46). Consistent with previous studies, we found that the proportion of the repertoire occupied by the most numerous 0.1% of T cell clones in the peripheral blood is dramatically increased in CMV-seropositive individuals across a wide range of ages. Although we did not examine study subjects longitudinally, our results from 543 CMV-seropositive and -seronegative individuals suggests that at the clonal level, “memory inflation” is a phenomenon that stabilizes; specifically, memory T cell clones reactive to persistent viruses do not continue to accumulate with increasing age. These results are supported by studies showing that in the elderly, CMV-specific T cells retain effective antiviral and cytotoxic functions (47–49). Nevertheless, these results underscore the substantial burden that CMV infection places on the aging adaptive immune system.

To explore the influence of CMV on the structure of the aging T cell repertoire in greater detail, we examined productively rearranged TCRβ DNA sequences derived from the CD8^+^ T cell subsets implicated in suppressing CMV reactivation in a group of eight CMV^+^ and CMV^−^ subjects aged 70 y and older. We found that the most numerous 0.1% of peripheral blood T cell clones composed a significant proportion of the memory and T_EMRA_ subsets in the CMV^+^ elderly, which is consistent with previous studies (41, 50). Given that a significant proportion of the memory and T_EMRA_ repertoires were composed of large clones in the CMV^+^ elderly, we next sought to determine whether these clones were CMV reactive. We found that, in both subjects, the largest CMV-reactive clones were shared among Ag-experienced T cell subsets. Significantly, we observed that some of the largest shared clones in the T_EMRA_ subset did not express CD137 when stimulated with CMV-infected fibroblasts. Our results are somewhat in conflict with another study in elderly individuals demonstrating that NLVPMVATV- and TPRVTGGGAM-specific T cells proliferate when stimulated with cognate peptide (47); however, the use of synthetic peptide pools opposed to CMV-infected fibroblasts as sources of Ag partially explains this discrepancy. Our observations are in agreement with previous reports that T_EMRA_ cells are not clonally deleted upon replicative senescence (51). Importantly, we found that the largest clones in the peripheral blood repertoire are CMV reactive. Considering that all participants were EBV seropositive and EBV is also known to elicit large clonal expansions, we suspect that some of the remaining large clones that are not specific to CMV could recognize EBV (52).

Previous studies have suggested that T cell clones are eradicated from the repertoire to accommodate the large clonal expansions observed in the CMV-seropositive elderly. However, we found no difference in the diversity of the underlying T cell repertoire or in the distribution of low-frequency clones in the naive T cell repertoire based on CMV serostatus (53). Our observations suggest that, from the standpoint of the CD8^+^ repertoire, CMV-driven clones expand without altering the rest of the repertoire (Fig. 5C–E) (54). Given our depth of sampling, the fact that we could not detect a compensatory shrinking of the repertoire in the presence of CMV-induced expansions implies that this phenomenon occurs either rarely or not at all. When we bioinformatically removed the most numerous 0.1% of peripheral blood T cell clones from the peripheral blood, memory, and T_EMRA_ repertoires, we observed an increase in diversity, and in fact, the diversity of these modified repertoires was nearly identical to that observed in the repertoires of CMV^−^ subjects. This suggests that the expansion of large, shared memory and T_EMRA_ clones may be a general phenomenon of aging in the context of a latent viral infection. In support of this claim, we observed that the total number of T cells in each CD8^+^ T cell subset is increased in CMV^+^ individuals. Altogether, these results demonstrate that the T cell repertoire grows to accommodate the maintenance of a population of large CMV-reactive T cell clones.

Although CMV infection alters the T cell repertoire across a variety of ages, CMV is not associated with increased mortality until the later stages of life. We note that although our inferences are based on a few participants, the differences in repertoire structure based on CMV status in the elderly are quite unremarkable. Thus, our observations merit further research into the cellular mechanisms that perpetuate immunosenescence.

## Supplementary Material

Data Supplement
