# Supplementary material for: Cytomegalovirus Exposure in the Elderly Does Not Reduce CD8 T Cell Repertoire Diversity
Source: J Immunol. 2018 Dec 12;202(2):476–83. doi: 10.4049/jimmunol.1800217 (PMC6321841; doi:10.4049/jimmunol.1800217)
Supplement: Data Supplement [file JI_1800217.zip › JI_1800217_Supplemental_Material_1.pdf]

Supplement

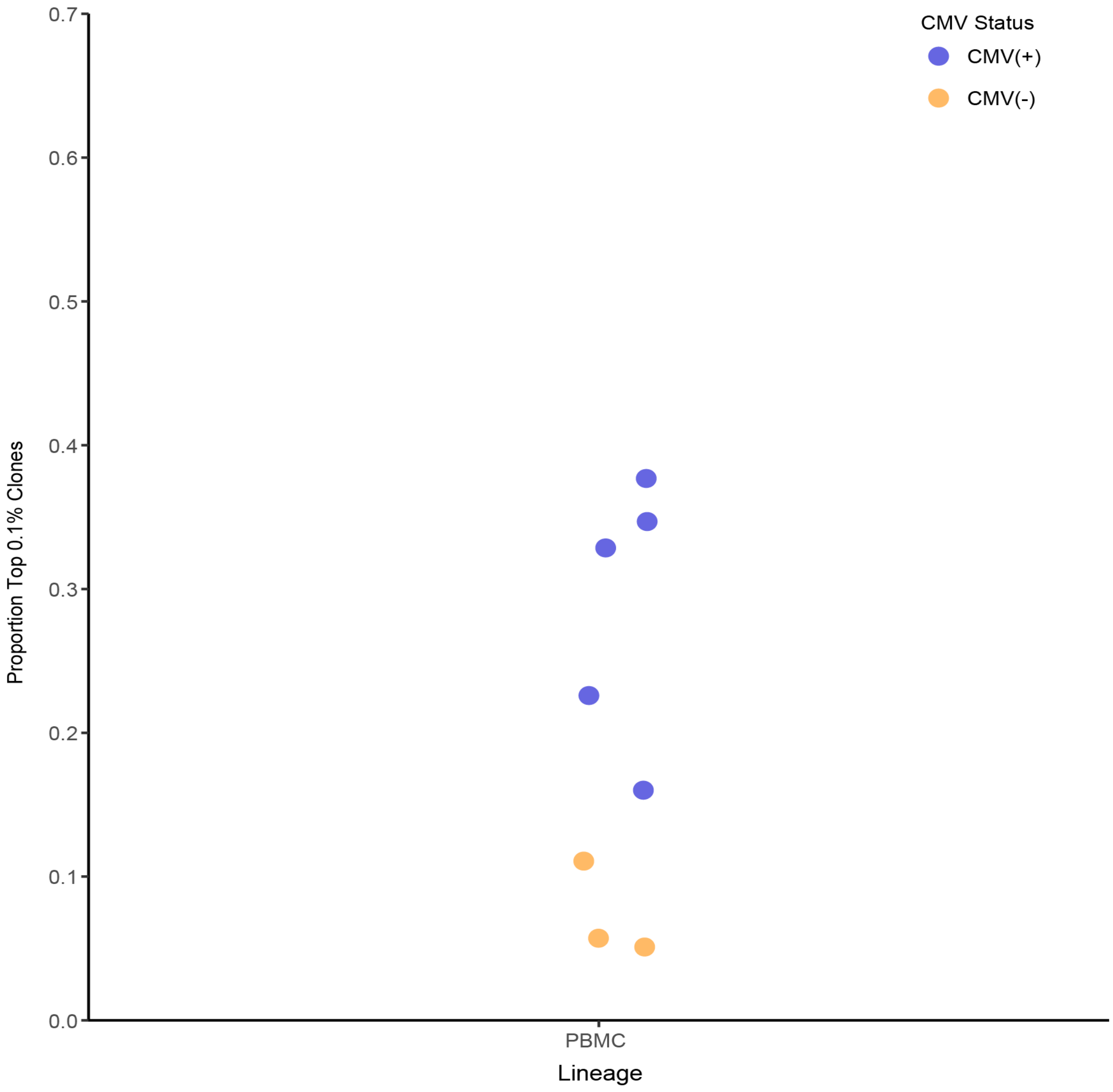

Figure. S1. **Most numerous 0.1% of peripheral blood T cell clones in 8 elderly subjects**. Scatterplot comparing the proportion of the most numerous 0.1% of clones in the peripheral blood T cell repertoires of 5 CMV^+ (blue) and 3 CMV^- (orange) elderly subjects. TCRβ chain sequencing was performed on the PBMCs of each subject. For each subject, the cumulative abundance of the most numerous 0.1% of clones was divided by the total sample abundance to yield a proportion. Non-productive TCRβ rearrangements were excluded from this calculation.

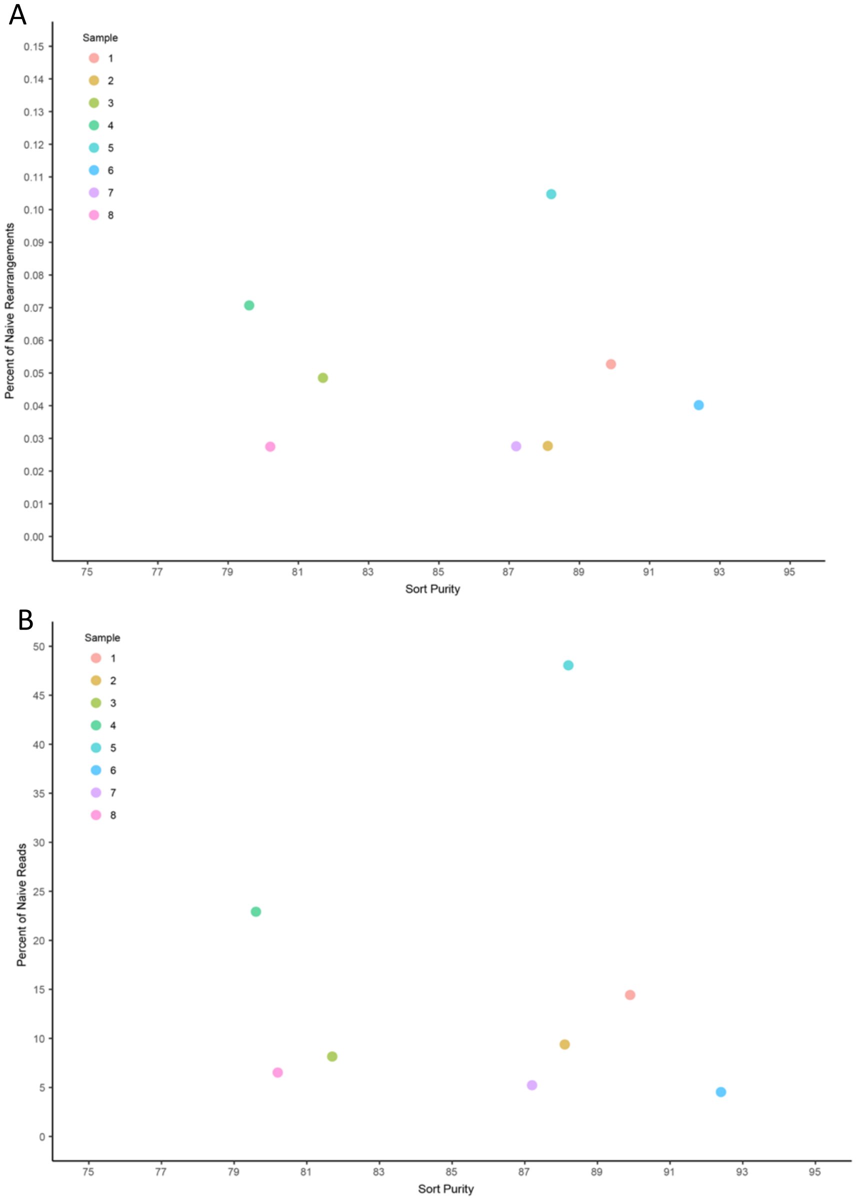

Figure. S2. **Contamination of the most numerous 0.1% of peripheral blood T cell clones in the naïve repertoire**. Comparison of the frequency of the most numerous 0.1% of peripheral blood T cell clones in the naïve repertoires of each elderly study subject. For each subject, the most numerous 0.1% of peripheral blood rearrangements present in the naïve repertoire are expressed **(A)** as a fraction of the total naïve repertoire rearrangements or **(B)** as a proportion of the total abundance of all clones in the naïve repertoire. Non-productive rearrangements are excluded from these calculations.

			CD8 Fraction (%)			Total Reads (10^6)				Total Rearrangements (10^4)			
Sample	CMV	Age	Naive	Memory	T_{EMRA}	PBMC	Naive	Memory	T_{EMRA}	PBMC	Naive	Memory	T_{EMRA}
1	(-)	71	40.78	42.12	17.00	10.42	5.93	9.74	3.21	17.18	21.73	6.11	1.56
2	(-)	70	55.46	32.42	12.10	13.27	9.47	12.90	0.57	11.28	25.28	1.33	1.47
3	(-)	72	29.40	61.40	9.17	14.02	16.09	13.04	19.46	14.59	22.05	3.57	4.60
4	(+)	71	23.31	51.32	25.40	6.12	3.01	7.17	10.75	13.61	9.68	3.68	0.90
5	(+)	73	19.60	57.65	22.80	9.45	3.67	8.19	13.10	18.16	7.63	2.62	0.33
6	(+)	73	25.21	61.12	13.70	7.21	7.03	15.08	17.12	12.05	23.89	2.42	2.22
7	(+)	70	18.11	45.12	36.80	7.36	10.41	15.16	16.70	7.71	22.84	2.72	3.52
8	(+)	74	33.01	41.80	25.20	3.21	12.54	9.52	5.04	9.93	26.21	2.68	1.26

Table S1. **Characteristics of 8 elderly study subjects**. Age, CMV status, sort purity and a summary of the immunosequencing data for each of the 8 elderly study subjects. The fraction of each $CD8^+$ subset was determined using flow cytometry. Only productively rearranged nucleotide sequences are counted in total rearrangements and sequencing reads for each subject.
